# Targeting an Oncolytic Influenza A Virus to Tumor Tissue by Elastase

**DOI:** 10.1016/j.omto.2017.09.002

**Published:** 2017-09-08

**Authors:** Irina Kuznetsova, Tobias Arnold, Thomas Aschacher, Cornelia Schwager, Balazs Hegedus, Tamas Garay, Marina Stukova, Maria Pisareva, Stephan Pleschka, Michael Bergmann, Andrej Egorov

**Affiliations:** 1 Department of Surgery, Medical University of Vienna, Währinger Gürtel 18-20, 1090 Vienna, Austria; 2 Avir Green Hills Biotechnology AG, Gersthoferstrasse 29, 1180 Vienna, Austria; 3 MTA-SE Molecular Oncology Research Group, Hungarian Academy of Sciences, Semmelweis University, Üllői út 93, 1091 Budapest, Hungary; 4 Research Institute of Influenza, Russian Academy of Medical Sciences, Prof. Popova Str. 15/17, 196376 St. Petersburg, Russia; 5 Institute for Medical Virology, Justus Liebig University Gießen, School of Medicine, Schubertstraße 81, 35392 Gießen, Germany; 6 Comprehensive Cancer Center, Medical University of Vienna, Währinger Gürtel 18-20, 1090 Vienna, Austria

**Keywords:** influenza A virus, neutrophilic elastase, oncolytic virotherapy, tumor immunology

## Abstract

Oncolytic viruses are currently established as a novel type of immunotherapy. The challenge is to safely target oncolytic viruses to tumors. Previously, we have generated influenza A viruses (IAVs) containing deletions in the viral interferon antagonist. Those deletions have attenuated the virus in normal tissue but allowed replication in tumor cells. IAV entry is mediated by hemagglutinin (HA), which needs to be activated by a serine protease, for example, through trypsin. To further target the IAV to tumors, we have changed the trypsin cleavage site to an elastase cleavage site. We chose this cleavage site because elastase is expressed in the tumor microenvironment. Moreover, the exchange of the cleavage site previously has been shown to attenuate viral growth in lungs. Newly generated elastase-activated influenza viruses (AE viruses) grew to similar titers in tumor cells as the trypsin-activated counterparts (AT viruses). Intratumoral injection of AE viruses into syngeneic B16f1 melanoma-derived tumors in mice reduced tumor growth similar to AT viruses and had a better therapeutic effect in heterologous human PANC-1-derived tumors. Therefore, the introduction of the attenuation marker “elastase cleavage site” in viral HA allows for safe, effective oncolytic virus therapy.

## Introduction

The main strategy of oncolytic viral therapy is based on the fact that naturally occurring or engineered viruses are able to conditionally infect tumor cells and replicate in them, thereby inducing cell lysis.^1^ The viral tropism to malignant cells is usually dependent on molecular alterations in cancer cells acquired during carcinogenesis, which leads to the loss of viral defense mechanisms, thus enabling viral amplification.^2^ These alterations of cancer cells involve defects in the interferon (IFN), p53, and pRb pathways along with an activation of the Ras/Raf1/MEK/ERK pathway^3^, ^4^, ^5^ or alterations in vessel walls. A number of phase II and phase III clinical trials using vaccinia, herpes virus, or adenoviruses have indicated the beneficial results of virotherapy, calling for the development of less explored virus families to generate clinically applicable prototypes of oncolytic viruses.^6^

We have generated an oncolytic influenza A virus (IAV) using the above-mentioned principles of molecular targeting of the virus to cancer cells. Specifically, we have shown that an attenuated vaccine prototype virus which lacks full-length NS1 protein, the viral antagonist of PKR,^7^ efficiently replicates in vitro in IFN-defective^8^ and *RAS* mutant cancer cells.^9^ Correspondingly, this prototype was effective as an oncolytic agent in different models in vivo.^8^ Continuous passaging of the oncolytic IAV resulted in adaptive mutations throughout the viral genome, which led to enhanced stability and growth for both tumor and producer cell lines without affecting pathogenicity.^10^

We hypothesized that the modification of the cleavage site of the hemagglutinin (HA), the viral entry protein, might further promote a conditionally replicative growth in tumors. The cleavage of HA by host proteases is an essential step in the life cycle of IAV, allowing multi-cycle replication and viral spread within infected tissue. All IAVs containing a monobasic cleavage site (a single Arg or Lys residues) require the presence of trypsin or trypsin-like serine proteases for activation.^11^, ^12^ Such proteases are typically found in the upper respiratory tract as well as in the gastrointestinal tract. For this reason, IAV infection is primarily limited to these sites. It was demonstrated that an IAV containing an elastase cleavage site in its HA is attenuated in mice and pigs.^13^, ^14^, ^15^, ^16^, ^17^ Thus, an alteration of HA protease susceptibility can attenuate viral spread.^18^ Importantly, malignant tissue is known to express proteases which promote tumor invasion. These proteases are either expressed by invading immune cells, such as neutrophils and macrophages, or by tumor cells themselves. They form a protease-rich environment around those malignant cells facilitating activation of oncolytic viruses.^19^

In this study, we investigated whether an attenuated IAV with an elastase-sensitive HA cleavage site is able to replicate in tumor cells and exerts an oncolytic effect in vivo. For this, we modified the HA of a previously generated GFP-expressing tumor adapted influenza A NS_116_-GFP/A virus.^10^ This alteration did not change viral growth characteristics in vitro when the suitable protease was added and multi-cycle viral replication remained when infected cells were co-cultivated with isolated neutrophils. Intratumoral application of the generated virus had an efficient therapeutic effect when different tumor models were used.

## Results

**Figure 1:**
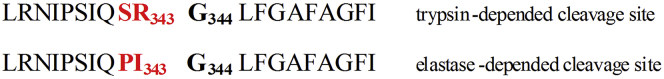
Schematic Representation of Modifications in the HA Cleavage Site The cleavage site is indicated by bold letters. The amino acids changed during adaptation to elastase are shown in red.

**Figure 2:**
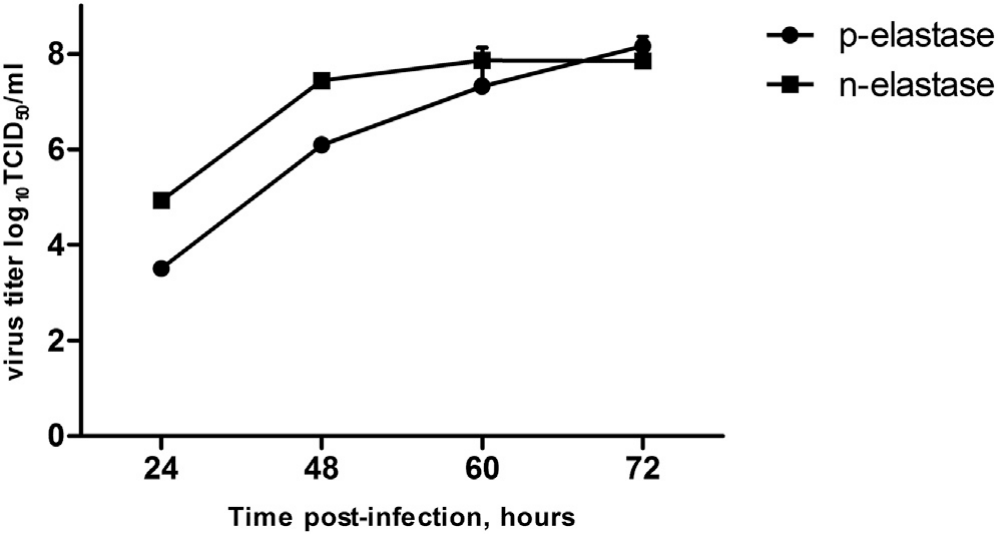
Growth Kinetic of Modified NS_116_-GFP/AE Virus A 24-hr-old monolayer of Vero cells was infected with NS_116_-GFP/AE virus in the presence of either pancreatic elastase (p-elastase) or neutrophil elastase (n-elastase). A viral titer was determined by TCID_50_ in Vero cells at the indicated time points + SEM (n = 6).

To obtain an elastase-dependent viral vector, we took advantage of the high mutability of influenza viruses and their ability to adapt. A trypsin-dependent influenza ΔNS1-H1N1 virus^20^ expressing an HA from influenza A/New Caledonia/20/99 virus was serially passaged on Vero cells in the presence of porcine pancreatic elastase. After 6 passages, we obtained a virus ΔNS1-H1N1-E, which could grow to a titer of 7 log 50% tissue culture infective dose (TCID_50_/ml) on Vero cells in the presence of porcine pancreatic elastase. Interestingly, the resulting virus was also sensitive to human neutrophil elastase activation (Figure S1). Sequence analysis of the HA gene revealed three nucleotide changes at positions 1056, 1060, and 1061 from T, G, A to C, T, G, respectively. Those changes corresponded to two amino acid substitutions at the cleavage site of the HA molecule: serine and arginine at positions 342 and 343 were replaced by proline and isoleucine, respectively (Figure 1). These mutations were then introduced into the genome of the previously described NS_116_-GFP/A^10^ virus by side-directed mutagenesis of the HA coding plasmid using standard reverse genetics methods developed for influenza virus.^21^, ^22^ Obtained mutant was named NS_116_-GFP/AE and was sensitive to activation by porcine pancreatic elastase and human neutrophil elastase (Figure 2). The parental virus which is activated by trypsin was named “NS_116_-GFP/AT.” We used this virus, as it has previously been shown to serve as a stable viral vector. Moreover, NS116-GFP/AT did not show any toxicity when systemically administrated by the intravenous route at a titer of 10^8^ TCID_50_ in mice (Figure S2). No infectious virus was isolated from brain, lungs, liver, gut, muscle, or kidney, indicating the lack of virus dissemination after systemic administration. Since a significant attenuation of elastase-dependent influenza virus in comparison with trypsin-dependent virus in mice model has been already shown,^15^, ^17^ we did not perform further biodistribution assays.

**Figure 3:**
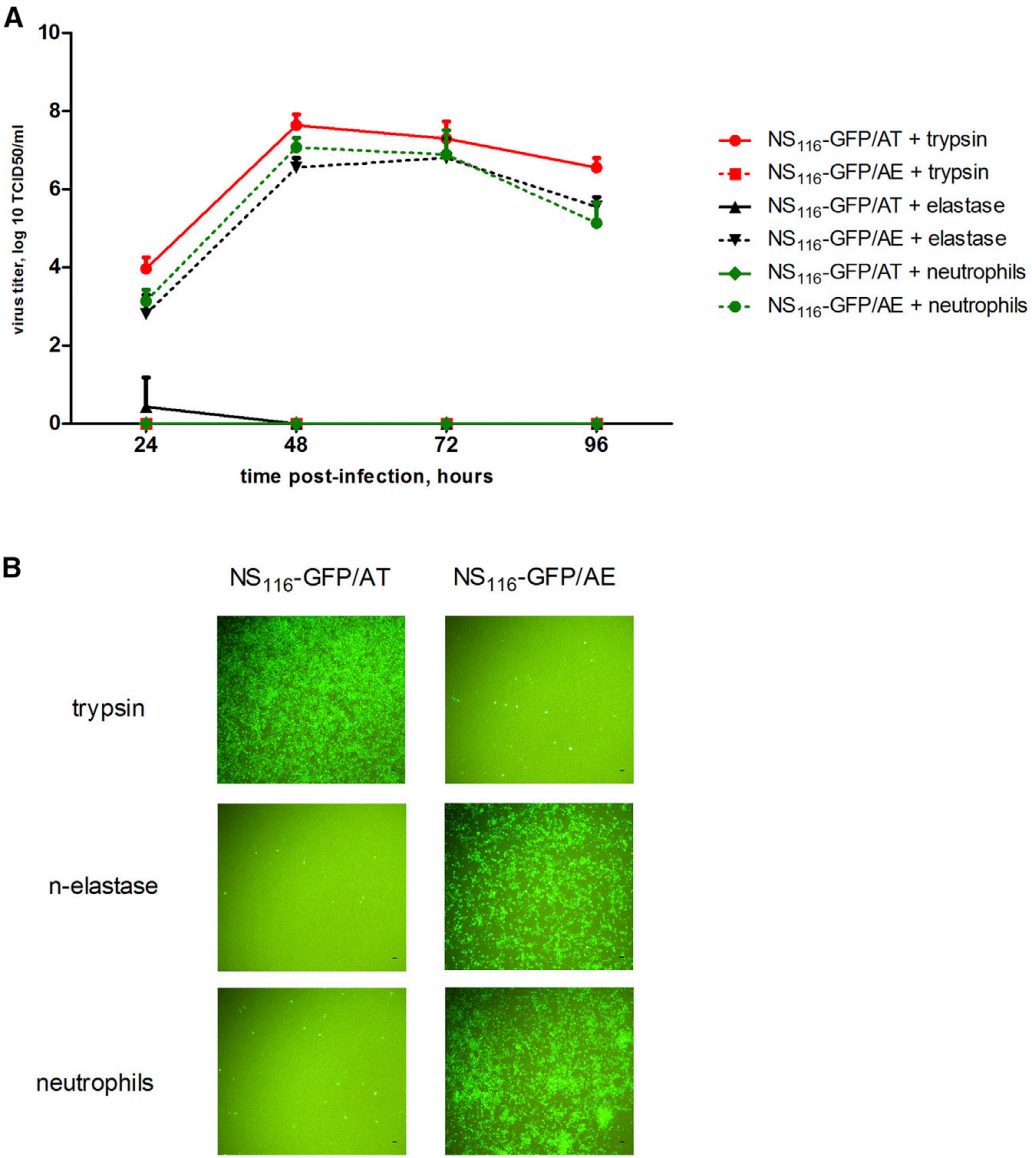
Growth Properties of NS_116_-GFP/AE and NS_116_-GFP/AT Virus in Vero Cells in Co-culture System with Human Neutrophils or Supernatant Supplemented with Neutrophil Elastase or Trypsin (A) Vero cells were infected at an MOI of 0.01 under indicated condition and viral titer was determined by TCID_50_ 24 hr post-infection. Data represent means + SEM (n = 3). (B) Virus induced GFP expression, which indicates viral growth. The pictures of infected Vero cells were taken 48 hr post-infection using an AxioCam ICc3 Rev.2-3 camera, with a magnification of 200×. Experiments were carried out three times with neutrophils of another proband, and the corresponding results are provided. Scale bar, 100 μm.

Next, we compared the protease-specificity of viruses NS_116_-GFP/AE and NS_116_-GFP/AT for trypsin, neutrophilic elastase or supernatant of human neutrophils. For later purposes, we determined the viral yield in a co-cultivation system of infected Vero cells with human neutrophils. The NS_116_-GFP/elastase-activated influenza virus (AE virus) grew to the same titers in the presence of neutrophils and in the presence of neutrophilic elastase, which were 7.06 log TCID_50_/ml and 6.8 log TCID_50_/ml, respectively (Figure 3A). This virus completely lost its reproductive activity in the presence of trypsin. In contrast, the NS_116_-GFP/AT virus grew to the titer of 7.6 log TCID_50_/ml in the presence of trypsin, but not in the presence of neutrophilic elastase or supernatants of neutrophils, indicating the specificity of the cleavage site. These titers were in correlation with the GFP expression in infected cells (Figure 3B). Our findings support the hypothesis that tumor-infiltrating immune cells, such as neutrophils, would produce elastase that is sufficient for HA cleavage of the NS_116_-GFP/AE virus replicating in tumor cells during oncolytic therapy.

**Figure 4:**
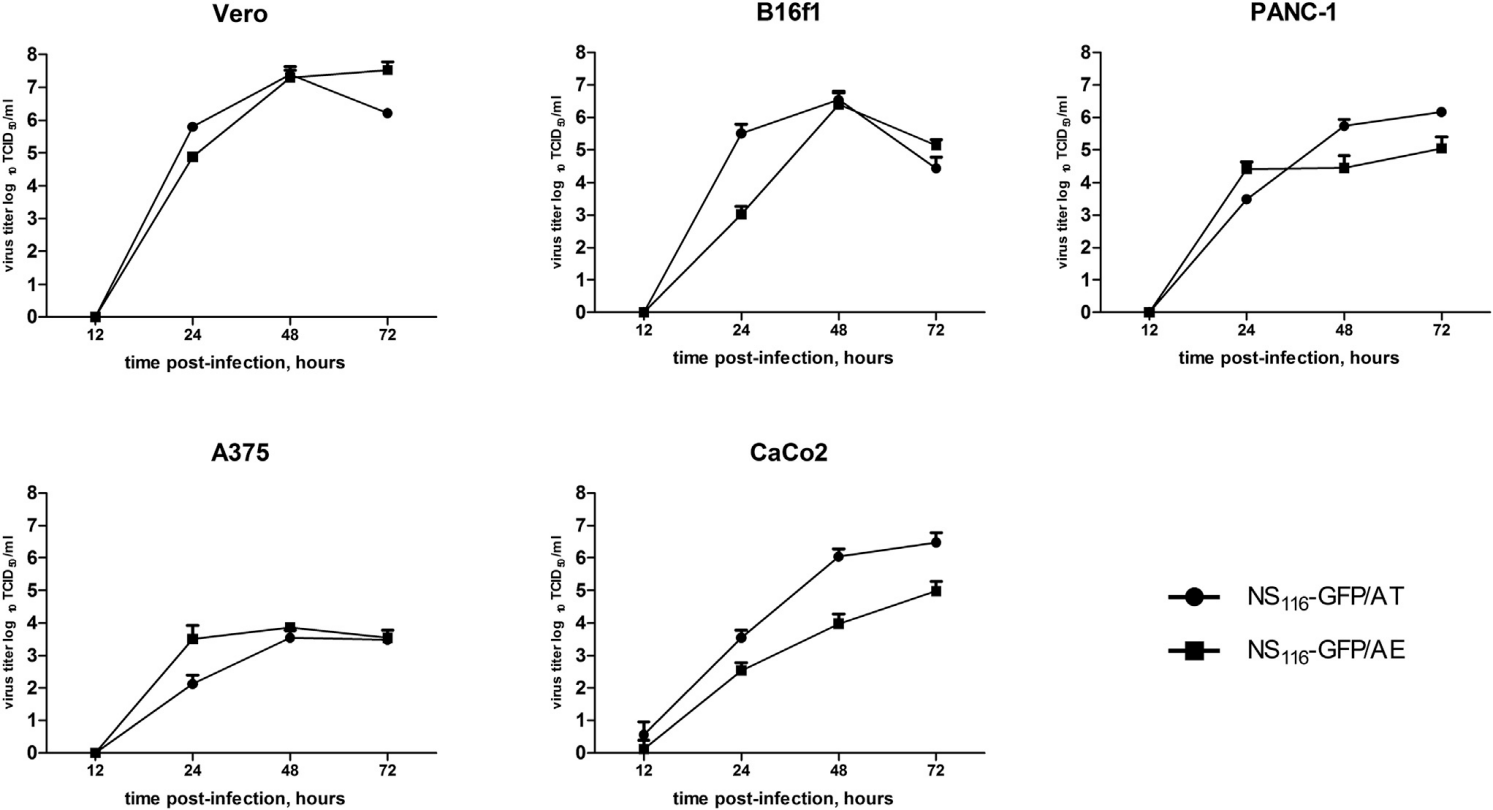
Growth of NS_116_-GFP/AT and NS_116_-GFP/AE Viruses in Different Cell Lines Vero, B16f1, PANC-1, A375, or CaCo2 1 × 10^5^ cells were infected with either NS_116_-GFP/AT or NS_116_-GFP/AE viruses at an MOI of 0.01 and cultivated in the presence trypsin or neutrophil elastase, respectively. Supernatant was collected 12, 24, 48, and 72 hr post-infection. Data represent means + SEM (n = 3).

To further address the growth properties of the elastase activated virus, we compared multi-cycle growth of NS_116_-GFP/AE and NS_116_-GFP/AT in Vero and B16f1 cells in the presence of neutrophilic elastase or trypsin, respectively. Change of the cleavage site in HA protein had no influence on the growth characteristic between the two viruses (Figure 4). Also, the modification of the HA-cleavage site did not lead to the growth disadvantages of NS_116_-GFP/AE virus in human tumor cell lines, such as PANC-1 or A375 (Figure 4). Trypsin-sensitive NS_116_-GFP/AT virus showed significantly higher titer in comparison with elastase-sensitive NS_116_-GFP/AE virus on the CaCo2 cell line. This might be explained by the endogenous production of trypsin-like serine proteases in this cell line allowing HA cleavage^17^ (Figure 4). None of the tested cell lines were able to support the replication of NS_116_-GFP/AE virus without a source of exogenous elastase (Figure S3). As expected the NS_116_-GFP/AT virus could replicate only in CaCo2 cells without an addition of trypsin (Figure S3).

**Figure 5:**
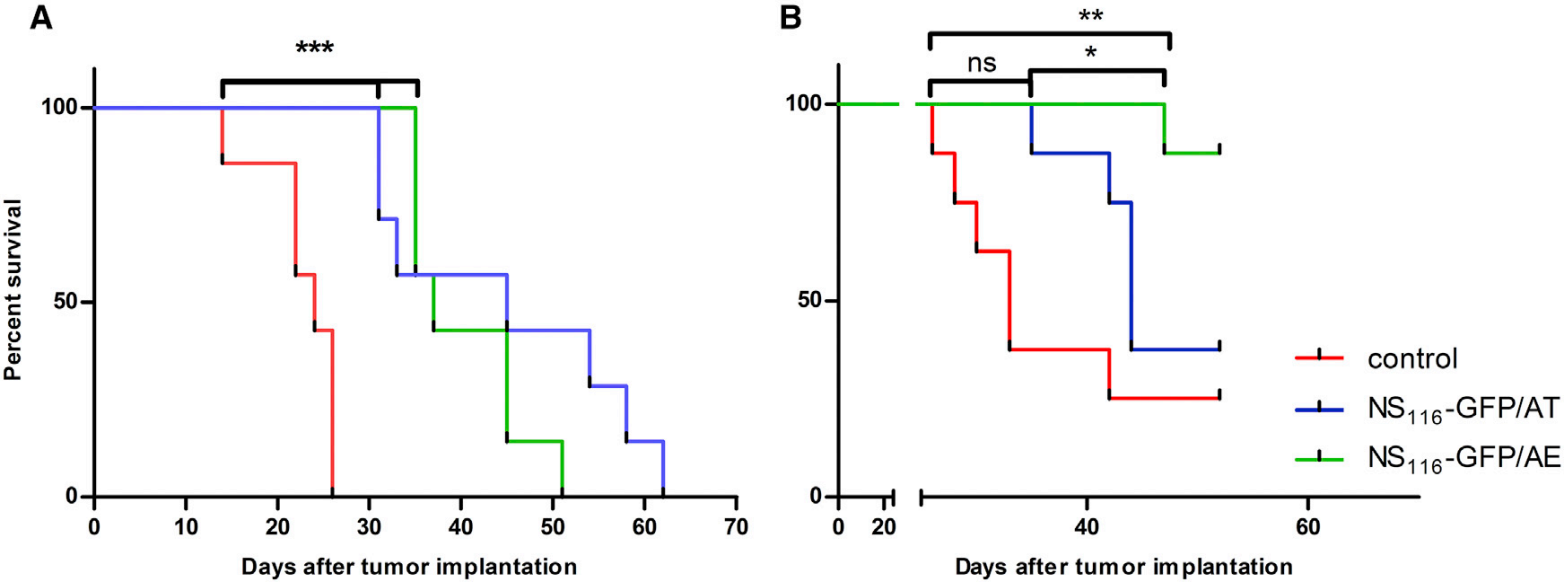
Effect of NS_116_-GFP/AT and NS_116_-GFP/AE Treatment on the Survival of B16f1 Melanoma- and PANC-1-Bearing Mice (A and B) Seven 6-week-old C57/BL6 or athymic nude mice were subcutaneously injected with B16f1 (A) or PANC-1 (B) cells. 5 days after implantation of melanoma cells and 10 days after implantation of pancreatic adenocarcinoma cells, the treatment with either NS_116_-GFP/AT and NS_116_-GFP/AE virus was started with the intratumoral injection of 1 × 10^7^ TCID_50_/mouse. Treatment was repeated on days 7, 9, 11, and 13 for C57/BL6 mice and on days 12, 14, 16, and 18 for PANC-1-bearing mice. The dose of the oncolytic virus was increased up to 1 × 10^8^ TCID_50_ /mouse for the last two injections. Mice were sacrificed when tumor volumes reached 2000 mm^3^. ns, not significant. *p < 0.05, **p < 0.01.

To test the oncolytic potential of NS_116_-GFP/AT and NS_116_-GFP/AE, we intratumorally injected these viruses into a syngeneic murine B16f1 model (Figure 5A). Administration of both, NS_116_-GFP/AT or NS_116_-GFP/AE viruses significantly inhibited tumor outgrowth, when compared to the control group (p < 0.05). There was no substantial difference between the two viruses. ΔNS1-H1N1 virus, a corresponding replication-deficient deltaNS1 virus lacking the complete NS1 open reading frame, had no therapeutic effect in this model (Figure S4). This complete NS1 deletion virus did not grow in B16f1 cells, indicating the critical role of virus replication for oncolytic activity.

**Figure 6:**
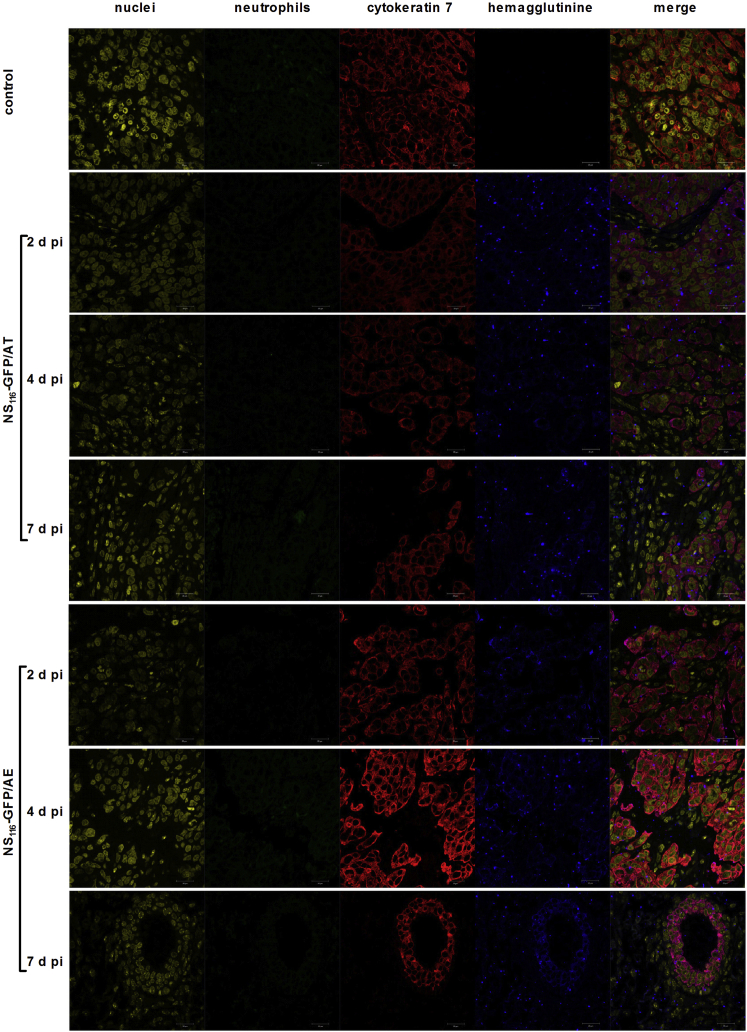
Detection of Viral Proteins and Neutrophil Infiltration in Tumor Tissue 2, 4, and 7 days after the first injection of either NS_116_-GFP/AT or NS_116_-GFP/AE virus, PANC-1 tumors were extracted and fixed in 3.7% formaldehyde. Immunohistochemical staining of de-paraffinized sections was performed using anti-hemagglutinin antibody (blue), anti-neutrophil antibody (green), anti-cytokeratin-7 antibody (as a marker of PANC-1 cells) (red), and DNA dye DRAQ5 (yellow). Scale bar, 20 μm.

The pancreatic cell line PANC-1 was chosen to study the oncolytic effect of the NS_116_-GFP/AE virus in a human tumor xenograft model (Figure 5B). It had been previously shown that influenza virus has an oncolytic activity in pancreatic cell lines.^23^ Interestingly, despite both viruses growing to comparable titers in PANC-1 cells in vitro, the NS_116_-GFP/AE virus had a significantly better therapeutic effect in this model compared to the NS_116_-GFP/AT virus (p < 0.05). We next determined the presence of influenza virus in tumor tissue by immunohistochemistry (Figure 6). This analysis indicated that both viruses replicated 7 days post-virus injections in the malignant cells as shown by the overlapping staining pattern of HA-specific and tumor-cell-specific signals. We did not observe a positive staining signal for neutrophils. This suggests that virus-activating elastase might have been produced by other cellular sources.

## Discussion

The use of potentially pathogenic viruses for oncolytic purposes calls for the development of multiple attenuation markers within the virus, which do not inhibit efficient growth in malignant cells. In IAV, we have previously defined such a marker when deleting a nonstructural protein NS1 coded by segment 8. This study examined another attenuation characteristic for conditional growth in tumors. An altered cleavage site of the hemagglutinin coded by segment 4 achieved the attenuation. Similar to the marker “NS1 deletion,” the marker “elastase cleavage site” attenuates the virus in the upper respiratory tissue, as has been previously shown.^16^, ^24^ Using two different murine models, we demonstrated that the introduction of the elastase cleavage site into the oncolytic influenza virus does not diminish the therapeutic properties of this oncolytic prototype in vivo. It should be noted that replication defective influenza viruses have little effect in tumor models, such as B16f1 melanoma (Figure S4). Thus, we conclude that the attenuation marker “elastase cleavage site” leads to a conditional replication of the virus in tumor tissue.

Pursuant to our in vitro data that neutrophilic elastase activated the elastase-dependent oncolytic virus, we initially hypothesized that tumor-infiltrating neutrophils could be the source for elastase in tumor tissue. However, we did not detect neutrophils in the PANC-1 tumor tissue where we observed a beneficial therapeutic effect of the elastase activated virus. Alternative sources of elastase could be macrophages^25^, ^26^ or tumor cells themselves. It was suggested that tumor cells have a “protease cloud” surrounding their surface. This “protease cloud” was shown to activate reovirus.^19^ However, as tumor cells in culture do not activate the NS_116_-GFP/AE virus in the absence of exogenous protease, it appears to be clear that the source of elastase within such a “protease cloud” is provided or stimulated by cells of the tumor microenvironment. Thus, activation of oncolytic viruses by proteases appears to be a more general and valuable approach to ensure virus targeting to tumors. We have now adapted this approach for influenza virus.

Our data also support previous findings^23^ that pancreatic cancer might be an attractive target for influenza virus oncolytic therapy. This virolysis of pancreatic cells may be supported by (1) the inherent susceptibility of pancreatic tissue to influenza virus^23^ and (2) by the fact that PANC-1 as well as 90% of pancreatic cancers have a *KRAS* gene mutation,^27^ leading to a suppression of the antiviral PKR pathway. Pancreatic cancer tissue specifically might be a target for elastase-dependent influenza virus given that the pancreatic cancer microenvironment often expresses proteases including elastase.^28^

An attenuation marker in segment 4 is of specific interest for the safe clinical application of the IAVs. The segmented nature of the virus could allow the spread of the HA used by oncolytic viruses within the population over an antigenic shift. A modification of the cleavage site within the HA molecule would prevent such a spread. In the same line, the attenuation marker in the HA might better allow the use of HA subtypes which are currently not circulating. This may be of specific interest for the repetitive use of the virus, as the exchange of the HA subtype will prevent inhibition of the oncolytic virus by preexisting immunity. Thus, introduction of the modified HA cleavage site should allow a safe use of H5 and/or H7 subtype and might further support application of selected H1 subtypes in cases of concern.

Modulation of virus entry proteins to target viruses to cancer has previously been conducted with virus families other than orthomyxoviruses.^29^, ^30^, ^31^ In this study, we applied this principle to IAV. A disadvantage of this approach could be that virus-entry-protein-modified viruses may impair viral growth, making production difficult and cumbersome. However, we demonstrated that the elastase-dependent virus grew as efficiently as the trypsin-activated virus in Vero cells, which is the cell line licensed for use for virus production. This indicates that the production of such viruses appears less of a problem.

In conclusion, we defined an attenuation maker, which allows conditional replication of the influenza virus in malignant tissue. We believe that this marker might be substantial to allow the use of rare HA influenza subtypes for oncolytic purposes, as it increases the safety of the therapeutic virus. Given that pancreatic tissue expresses pancreatic elastase, oncolytic influenza viruses expressing an elastase cleavage site might be of specific interest for pancreatic cancer.

## Materials and Methods

### Cell Lines

All cell lines were purchased from the ATCC (ATCC, Manasses, VA, USA). Human adenocarcinoma cell line PANC-1, human melanoma cells A375, and mouse melanoma cells B16f1 were cultured in DMEM/nutrient mixture F12 (GIBCO, Life Technologies, Grand Island, NY, USA) supplemented with 10% fetal calf serum (FCS) (GIBCO). Adenocarcinoma cell line CaCo2 was maintained in Eagle’s Minimum Essential Medium (EMEM) (GIBCO) supplemented with 10% FCS. Monkey kidney epithelial (Vero) cells were cultured in serum-free OptiPRO medium (GIBCO).

### Viruses

The ΔNS1-H1N1 A/New Caledonia virus^20^ contains HA and NA from A/New Caledonia/20/99 (H1N1), PB1 of A/Texas/1/77 (H3N2), and PA, PB2, NP, M, and NS are of PR8 origin A/Puerto Rico/8/34 and were adapted by serial passages to grow on Vero cells in presence of 5 μg/ml porcine pancreatic elastase (Sigma-Aldrich, St. Louis, MO, USA). Trypsin-dependent NS_116_-GFP/AT or elastase-dependent NS1_116_-GFP/AE viruses were propagated at 37°C in Vero cells infected with MOI of 0.01 in the presence of either 1 μg L-1-Tosylamide-2-phenylethyl chloromethyl ketone (TPCK)-treated trypsin (Sigma Aldrich) ml^−1^ or 10 μg human neutrophil elastase (Serva Electrophoresis, Heidelberg, Germany) ml^−1^. Virus titer was determined by a TCID_50_ assay.

### Plasmids

Viral RNA of ΔNS1-H1N1 virus was isolated from 280 μl of supernatant of infected cells using a QIAamp Viral RNA Mini Kit (QIAGEN, Hilden, Germany), and cDNA was generated by using a Uni12 primer (5′-AGCAAAAGCAGG-3′)^21^ and Superscript II RT (Invitrogen, Carlsbad, CA, USA), according to the manufacturer’s protocol. The cDNA of the HA segment was amplified by PCR using segment-specific primers,^21^ and nucleotide-sequencing analysis was performed by GeneArt (Invitrogen). The revealed nucleotide changes within the sequence of the HA cleavage site T1056C, G1060T, and A1061G were introduced in pHW2000^22^ vector coding HA of NS_116_-GFP/A virus^10^ by site-directed mutagenesis using the primers 5′-CAT TCCGTCCATTCAACC CGC AGGTCTATT TGGAGC-3′, 5′- CAT TCC GTC CAT TCA ATC CGC AGG TCT ATT TGG AGC-3′, 5′- GCT CCA AAT AGA CCT GCG GGT TGA ATG GAC GGA ATG-3′, and 5′- GCT CCA AAT AGA CCT GCG GAT TGA ATG GAC GGA ATG-3′.

### Generation of the Viruses

The eight plasmid reverse genetic system^22^ was used to generate NS_116_-GFP/AE virus, as previously described.^10^ Briefly, 4 μg of pHW2000 plasmids encoding single-gene segments of NS_116_-GFP/AE were transfected into 1 × 10^6^ of Vero cells using the Nucleofector technique (Lonza, Basel, Switzerland). After transfection, cells were incubated in OptiPRO medium containing 10 μg/ml neutrophil elastase. When the cytopathic effect (CPE) reached 50%, supernatant was collected and two consecutive passages were performed on Vero cells before the virus stock was produced. The virus titer was determined by the TCID_50_ assay.

### Determination of Viral Replication Kinetic

0.1 × 10^6^ of A375, B16f1, Caco2, PANC-1, or Vero cells were seeded in 12-well plates, and subconfluent monolayers of the cells were infected the next day with the relevant virus at an MOI of 0.01. Cells were incubated in the presence of TPCK-treated trypsin, neutrophil elastase, or without any of proteases. The supernatant was collected at the indicated time points, and the TCID_50_ assay was exploited for the determination of virus titers.

### Isolation of Human Polymorphonuclear Leukocytes

The trial was approved by the Ethics Committee of the Medical University of Vienna (#455/2010). Human polymorphonuclear leukocytes (PML) were isolated from peripheral blood of healthy donors, as previously described.^32^ Briefly, fresh venous blood was loaded on Ficoll-Paque (Amersham Pharmacia Biotech, Uppsala, Sweden) and centrifuged at 350 × *g* for 35 min. PMLs were collected and separated from the red blood cells by 6% dextran 500 (Sigma Aldrich) sedimentation, followed by hypotonic lysis of the remaining erythrocytes.

### Co-cultivation of PMLs and Infected Vero Cells

0.5 × 10^6^ Vero cells seeded in 12-well plates were infected with either NS_116_-GFP/AT or NS_116_-GFP/AE virus at an MOI of 0.01 for 45 min at room temperature. Subsequent inoculum was removed, and cells were washed with PBS. Based on an estimated cellular amount of neutrophil elastase equal to 3 pg/cell,^33^, ^34^ 5 × 10^6^ of isolated PML was resuspended in 1.5 mL of OptiPRO medium and placed in Transwell insert chambers (Costar, New York, NY, USA), preventing direct contact between infected Vero and non-infected PMLs. As a control, 1.5 mL of OptiPRO medium containing either 1 mg/ml TPCK-trypsin or 10 μg/ml human neutrophil elastase was used. 24, 48, 72, and 96 hr post-infection supernatants were collected, and the virus titers were determined by TCID_50_ assay. The pictures of infected monolayers were taken by AxioCam ICc3 Rev.2-3 camera for a Zeiss Axiovert 40 CFL microscope (Zeiss, Jena, Germany).

### TCID_50_ Assay

For the TCID50 assay, Vero cells were seeded in 96-well plates at a concentration of 3 × 10^6^ cells/plate. The medium was removed the next day, and the cells were incubated for 45 min with 50 μl of 10-fold virus dilution in an OptiPRO medium. After incubation, 50 μl of OptiPRO medium containing 1 mg/ml trypsin was added to each well. The virus titer was calculated 48 hr later by the Reed and Muench method and presented as a log10 TCID_50_ per ml.

### In Vivo Treatment of Established Tumor

All animal experiments were performed according to the latest guidelines of the ‘‘Federation of European Laboratory Animal Science Associations (FELASA)” and approved by the local committee of the Medical University of Vienna (BMWF-66-009/0204-II/3b/2011). 6- to 8-week-old female mice were purchased from Charles River Laboratories (Sulzfeld, Germany). For the syngeneic tumor model, 5 × 10^5^ B16f1 cells resuspended in 100 μl of PBS were subcutaneously injected into the right groin of C57BL/6 mice (n = 7). For the heterologous tumor model, 3 × 10^6^ PANC-1 cells resuspended in 100 μl of PBS were injected in the right flank of athymic nude mice (n = 8). Administration of the therapeutic viral injections began 5 and 10 days after the tumors were visible and were ∼2–3 mm in diameter for B16f1 and PANC-1 cells, respectively. Tumor size was measured using digital calipers each second day, and tumor volume (V) was calculated using the formula $V=\left(\mathrm{\pi b}a^{2}/6\right)$, where ***a*** is the smallest diameter and ***b*** is the perpendicular diameter.

### Immunohistofluorescence

5-μm tissue sections of paraffin-embedded PANC-1 tumors were rehydrated and then stained for 1 hr with rabbit anti-cytokeratine-8 antibody diluted 1:100 (Abcam, Cambridge, UK) to detect PANC-1 cells; sheep anti-HA-antibody was diluted 1:200 to detect influenza-infected cells; and rat anti-mouse Ly-6B.2 was diluted 1:50 to detect neutrophils (AbD Seotech, Oxford, UK). Tissue sections were washed twice with PBS and incubated with secondary antibody: anti-rat Alexa Fluor 488, anti-rabbit Alexa Fluor 555, or anti-sheep Alexa Fluor 633 (Thermo Scientific, Waltham, MA, USA) for 1 hr, followed by washing with PBS and incubation with 5 μM DRAQ5 (Thermo Scientific) to stain nuclei. The images were acquired using Carl Zeiss LSM 700 confocal microscope.

### Statistical Analysis

Statistical analysis was performed by two-tailed Student t test, and Kaplan-Meyer survival curves were compared using the log-rank (Mantel-Cox) test (Prism 5, Graph Pad Software).

## Author Contributions

Conceptualization, A.E. and M.B.; Methodology, A.E., M.B., M.S., M.P., and B.H.; Investigation, I.K., T. Arnold, T. Aschacher, C.S., T.G, M.S., and M.P.; Writing-Original Draft, I.K., M.B., and A.E.; Writing – Review & Editing, I.K., M.B., A.E., S.P., and T. Arnold; Funding Acquisition, M.B. and A.E.; Resources, M.B., B.H., M.S., M.P., and S.P.; Supervision, M.B. and A.E.

## Conflicts of Interest

All authors declare no potential conflicts of interest.

## Supplemental Information

Document S1. Supplemental Materials and Methods and Figures S1–S4

Document S2. Article plus Supplemental Information

## References

[1] Everts B., van der Poel H.G. (2005). Replication-selective oncolytic viruses in the treatment of cancer. Cancer Gene Ther..

[2] Bartlett D.L., Liu Z., Sathaiah M., Ravindranathan R., Guo Z., He Y., Guo Z.S. (2013). Oncolytic viruses as therapeutic cancer vaccines. Mol. Cancer.

[3] Vähä-Koskela M.J., Heikkilä J.E., Hinkkanen A.E. (2007). Oncolytic viruses in cancer therapy. Cancer Lett..

[4] Guo Z.S., Thorne S.H., Bartlett D.L. (2008). Oncolytic virotherapy: molecular targets in tumor-selective replication and carrier cell-mediated delivery of oncolytic viruses. Biochim. Biophys. Acta.

[5] Dorer D.E., Nettelbeck D.M. (2009). Targeting cancer by transcriptional control in cancer gene therapy and viral oncolysis. Adv. Drug Deliv. Rev..

[6] Russell S.J., Peng K.W., Bell J.C. (2012). Oncolytic virotherapy. Nat. Biotechnol..

[7] Bergmann M., Garcia-Sastre A., Carnero E., Pehamberger H., Wolff K., Palese P., Muster T. (2000). Influenza virus NS1 protein counteracts PKR-mediated inhibition of replication. J. Virol..

[8] Muster T., Rajtarova J., Sachet M., Unger H., Fleischhacker R., Romirer I., Grassauer A., Url A., García-Sastre A., Wolff K. (2004). Interferon resistance promotes oncolysis by influenza virus NS1-deletion mutants. Int. J. Cancer.

[9] Bergmann M., Romirer I., Sachet M., Fleischhacker R., García-Sastre A., Palese P., Wolff K., Pehamberger H., Jakesz R., Muster T. (2001). A genetically engineered influenza A virus with ras-dependent oncolytic properties. Cancer Res..

[10] Kuznetsova I., Shurygina A.P., Wolf B., Wolschek M., Enzmann F., Sansyzbay A., Khairullin B., Sandybayev N., Stukova M., Kiselev O. (2014). Adaptive mutation in nuclear export protein allows stable transgene expression in a chimaeric influenza A virus vector. J. Gen. Virol..

[11] Zhirnov O.P., Ikizler M.R., Wright P.F. (2002). Cleavage of influenza a virus hemagglutinin in human respiratory epithelium is cell associated and sensitive to exogenous antiproteases. J. Virol..

[12] Böttcher-Friebertshäuser E., Freuer C., Sielaff F., Schmidt S., Eickmann M., Uhlendorff J., Steinmetzer T., Klenk H.D., Garten W. (2010). Cleavage of influenza virus hemagglutinin by airway proteases TMPRSS2 and HAT differs in subcellular localization and susceptibility to protease inhibitors. J. Virol..

[13] Stech J. (2008). Attenuated influenza A viruses with modified cleavage sites in hemagglutinin as live vaccines. Expert Rev. Vaccines.

[14] Masic A., Lu X., Li J., Mutwiri G.K., Babiuk L.A., Brown E.G., Zhou Y. (2010). Immunogenicity and protective efficacy of an elastase-dependent live attenuated swine influenza virus vaccine administered intranasally in pigs. Vaccine.

[15] Gabriel G., Garn H., Wegmann M., Renz H., Herwig A., Klenk H.D., Stech J. (2008). The potential of a protease activation mutant of a highly pathogenic avian influenza virus for a pandemic live vaccine. Vaccine.

[16] Masic A., Babiuk L.A., Zhou Y. (2009). Reverse genetics-generated elastase-dependent swine influenza viruses are attenuated in pigs. J. Gen. Virol..

[17] Stech J., Garn H., Wegmann M., Wagner R., Klenk H.D. (2005). A new approach to an influenza live vaccine: modification of the cleavage site of hemagglutinin. Nat. Med..

[18] Rumschlag-Booms E., Rong L. (2013). Influenza a virus entry: implications in virulence and future therapeutics. Adv. Virol..

[19] Alain T., Kim T.S., Lun X., Liacini A., Schiff L.A., Senger D.L., Forsyth P.A. (2007). Proteolytic disassembly is a critical determinant for reovirus oncolysis. Mol. Ther..

[20] Wacheck V., Egorov A., Groiss F., Pfeiffer A., Fuereder T., Hoeflmayer D., Kundi M., Popow-Kraupp T., Redlberger-Fritz M., Mueller C.A. (2010). A novel type of influenza vaccine: safety and immunogenicity of replication-deficient influenza virus created by deletion of the interferon antagonist NS1. J. Infect. Dis..

[21] Hoffmann E., Stech J., Guan Y., Webster R.G., Perez D.R. (2001). Universal primer set for the full-length amplification of all influenza A viruses. Arch. Virol..

[22] Hoffmann E., Webster R.G. (2000). Unidirectional RNA polymerase I-polymerase II transcription system for the generation of influenza A virus from eight plasmids. J. Gen. Virol..

[23] Capua I., Mercalli A., Pizzuto M.S., Romero-Tejeda A., Kasloff S., De Battisti C., Bonfante F., Patrono L.V., Vicenzi E., Zappulli V. (2013). Influenza A viruses grow in human pancreatic cells and cause pancreatitis and diabetes in an animal model. J. Virol..

[24] Masic A., Booth J.S., Mutwiri G.K., Babiuk L.A., Zhou Y. (2009). Elastase-dependent live attenuated swine influenza A viruses are immunogenic and confer protection against swine influenza A virus infection in pigs. J. Virol..

[25] Nenan S., Boichot E., Lagente V., Bertrand C.P. (2005). Macrophage elastase (MMP-12): a pro-inflammatory mediator?. Mem. Inst. Oswaldo. Cruz..

[26] Houghton A.M., Grisolano J.L., Baumann M.L., Kobayashi D.K., Hautamaki R.D., Nehring L.C., Cornelius L.A., Shapiro S.D. (2006). Macrophage elastase (matrix metalloproteinase-12) suppresses growth of lung metastases. Cancer Res..

[27] Watanabe M., Nobuta A., Tanaka J., Asaka M. (1996). An effect of K-ras gene mutation on epidermal growth factor receptor signal transduction in PANC-1 pancreatic carcinoma cells. Int. J. Cancer.

[28] Lowe A.W., Olsen M., Hao Y., Lee S.P., Taek Lee K., Chen X., van de Rijn M., Brown P.O. (2007). Gene expression patterns in pancreatic tumors, cells and tissues. PLoS ONE.

[29] Cattaneo R., Miest T., Shashkova E.V., Barry M.A. (2008). Reprogrammed viruses as cancer therapeutics: targeted, armed and shielded. Nat. Rev. Microbiol..

[30] O’Shea C.C. (2005). Viruses - seeking and destroying the tumor program. Oncogene.

[31] Singh P.K., Doley J., Kumar G.R., Sahoo A.P., Tiwari A.K. (2012). Oncolytic viruses & their specific targeting to tumour cells. Indian J. Med. Res..

[32] Oehler R., Weingartmann G., Manhart N., Salzer U., Meissner M., Schlegel W., Spittler A., Bergmann M., Kandioler D., Oismüller C. (2000). Polytrauma induces increased expression of pyruvate kinase in neutrophils. Blood.

[33] Liou T.G., Campbell E.J. (1995). Nonisotropic enzyme--inhibitor interactions: a novel nonoxidative mechanism for quantum proteolysis by human neutrophils. Biochemistry.

[34] Kawabata K., Hagio T., Matsuoka S. (2002). The role of neutrophil elastase in acute lung injury. Eur. J. Pharmacol..

