# Supplementary material for: Targeting an Oncolytic Influenza A Virus to Tumor Tissue by Elastase
Source: Mol Ther Oncolytics. 2017 Sep 8;7:37–44. doi: 10.1016/j.omto.2017.09.002 (PMC5633860; doi:10.1016/j.omto.2017.09.002)
Supplement: Document S1. Supplemental Materials and Methods and Figures S1–S4 [file mmc1.pdf]

OMTO, Volume *7*

Supplemental Information

Targeting an Oncolytic Influenza A Virus to Tumor Tissue by Elastase

Irina Kuznetsova, Tobias Arnold, Thomas Aschacher, Cornelia Schwager, Balazs Hegedus, Tamas Garay, Marina Stukova, Maria Pisareva, Stephan Pleschka, Michael Bergmann, and Andrej Egorov

Supplemental Figures.

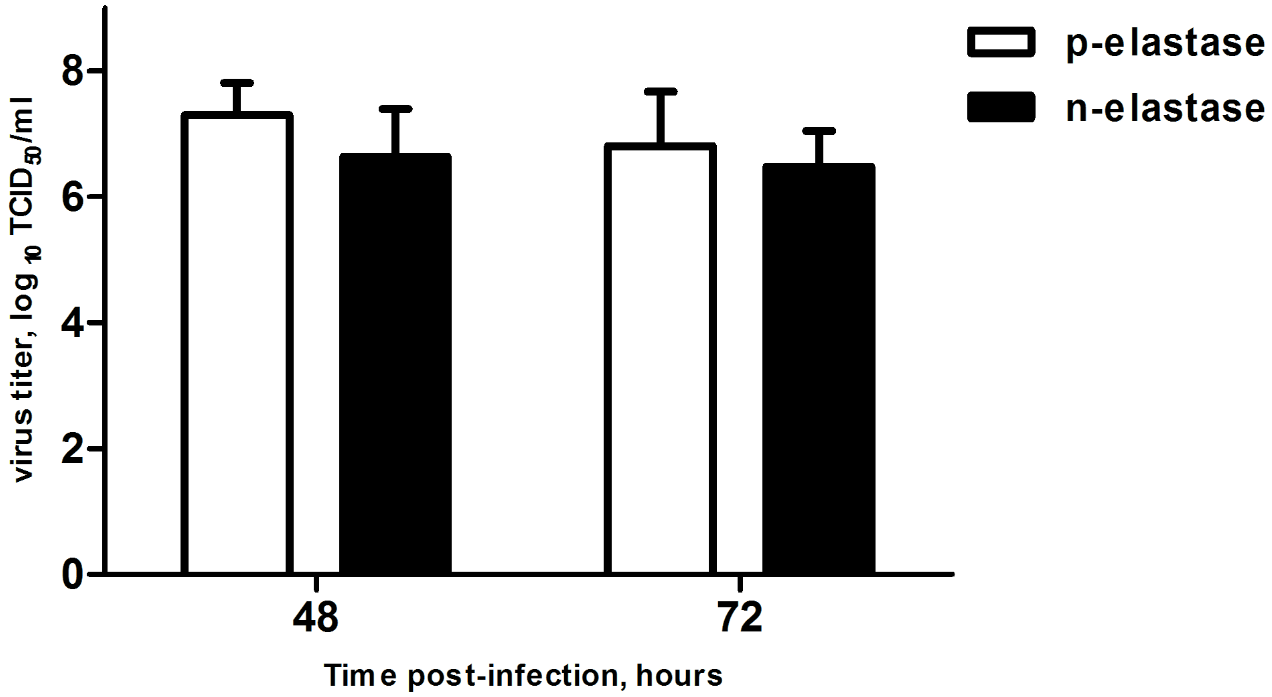

Figure S1. Growth properties of ΔNS1-H1N1-E virus. 24 hours-old monolayer of Vero cells was infected with ΔNS1-H1N1 virus at an MOI 0.01 and cultivated in the presence of either pancreatic elastase or neutrophil elastase. Viral titer was determined by $TCID_{50}$ assay at the indicated time points (+SEM, n=6)

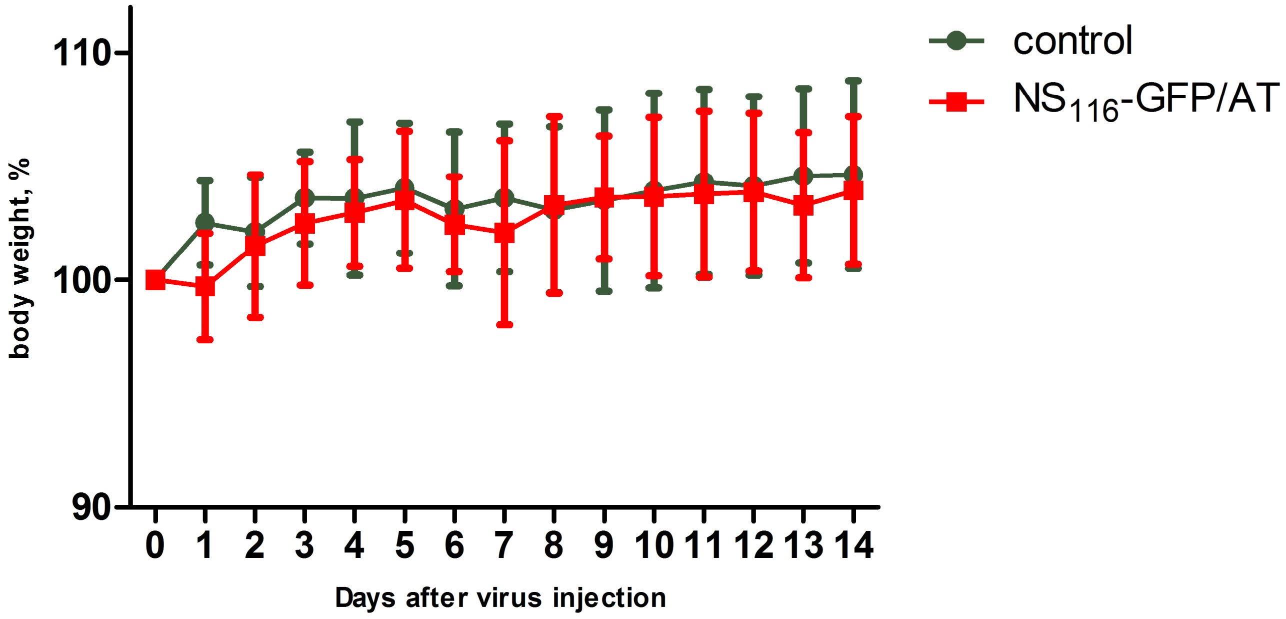

Figure S2. Pathogenicity of NS_{116} - GFP/AT virus in mice. 100µl of NS_{116} - GFP/AT virus or buffer was applied to mice (n=10/ group) intravenously (iv). Body weight of virus was measured daily up to 14 days as indicated. (±SEM, n=10).

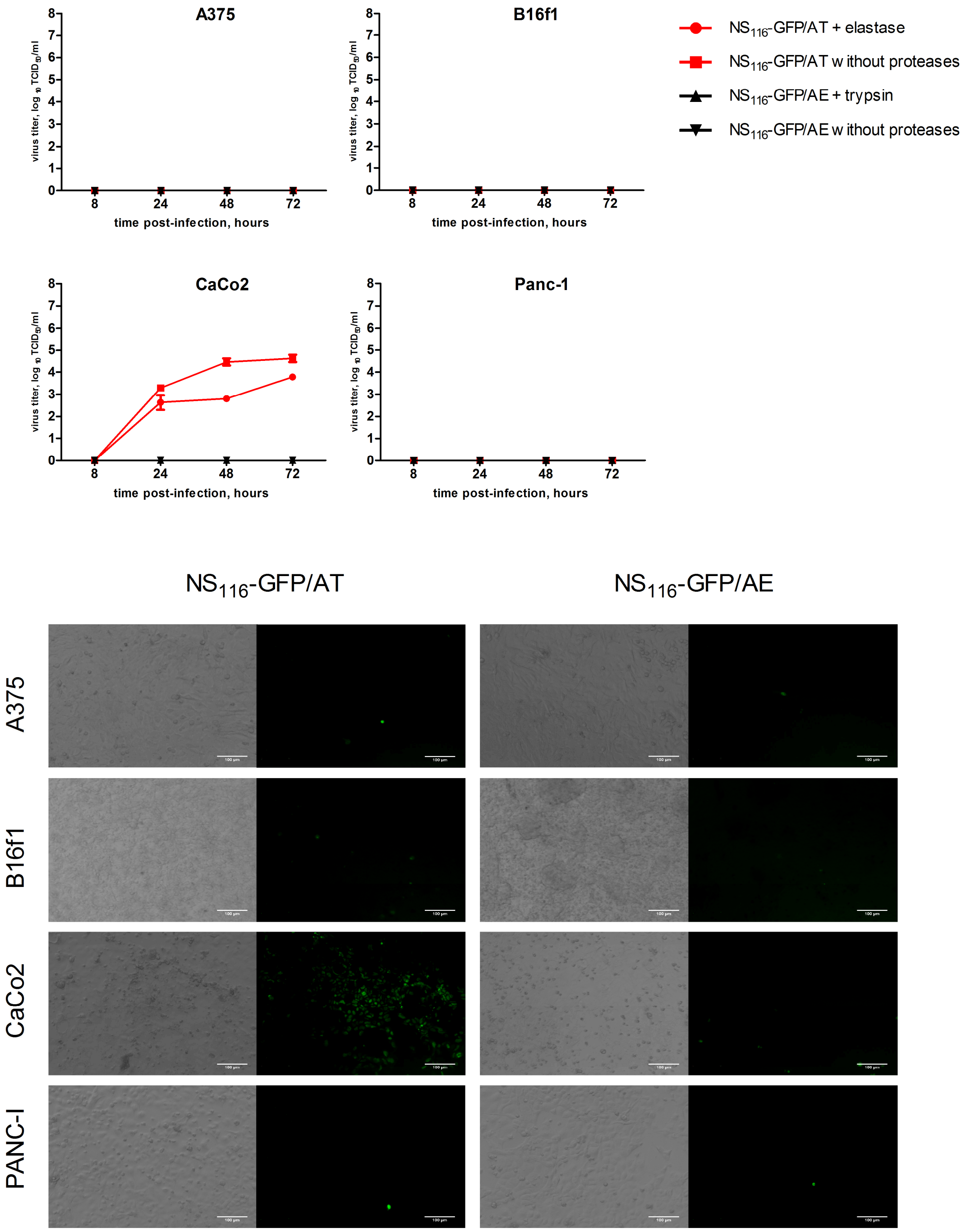

Figure S3. Growth kinetic of NS_{116}-GFP/AT and NS_{116}-GFP/AE viruses in different cell lines without exogenous proteases. A375, B16f1, CaCo2 and Panc-1 cells were infected with either NS_{116}-GFP/AT or NS_{116}-GFP/AE viruses at an MOI 0.01 and cultivated in the presence of neutrophil elastase or trypsin respectively, or without any of proteases. Virus titers in supernatant of infected cells at indicated time points were determined by $TCID_{50}$ assay (+SEM, n=6). Scale bar = 100 µm

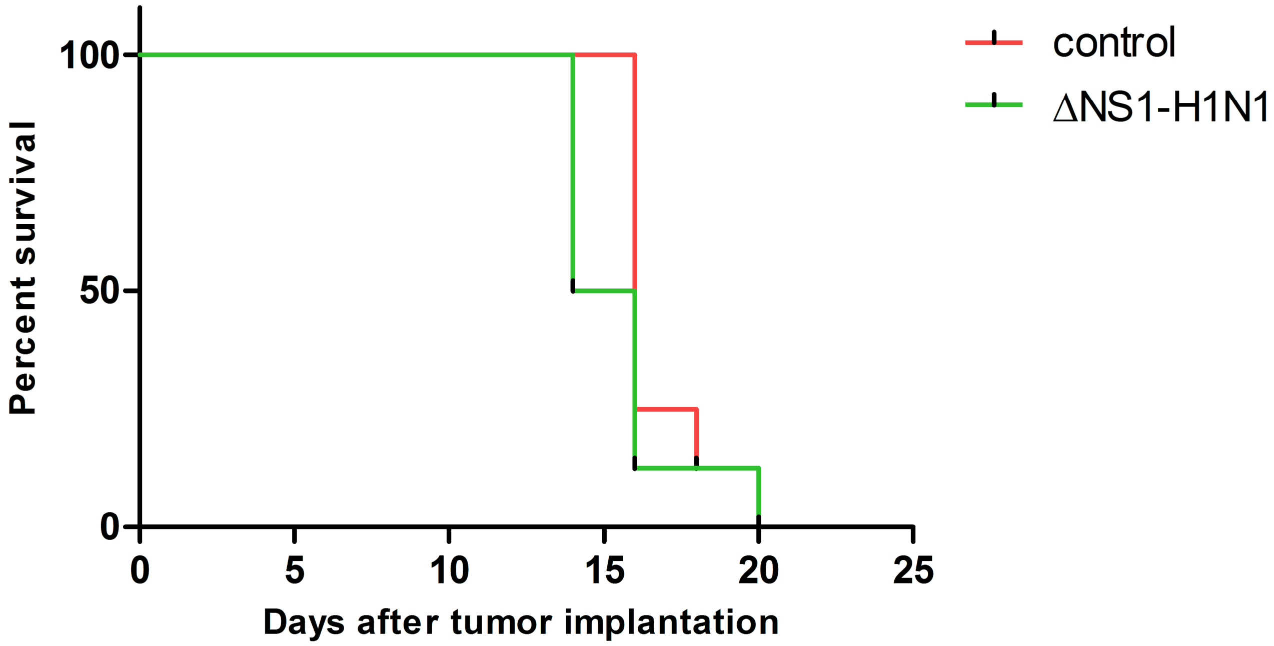

Figure S4. Survival of B16f1 melanoma-bearing mice treated with ΔNS1-H1N1 virus. C57/BL6 mice were treated with ΔNS1-H1N1 virus by intratumoral injection on the day 5, 7, 9 and 11 after tumor implantation. Single injection contained $1x10^7$ $TCID_{50}$ of virus particles.

Supplemental Methods and Materials.

Biodistribution assay. Toxicity study of IAV-AT virus was performed using Balb/c mice (n=10). The virus was applied by intravenous injection of 100 µl of virus suspension containing 8 logs of TCID50 of virus. Body weight was measured each day within 14 days period. As a control 100 µl of 0.9% NaCl was used. For a bio-distribution assay 5 animals per group have been tested. On the day 2 and day 4 mice were sacrificed, brain, lungs, heart, kidney, liver, intestinum, spleen excised and rinsed briefly in PBS. Organs were homogenized in RPMI1640 medium (Gibco) supplemented with penicillin (100 U/mL) (Gibco), streptomycin (100 U/mL) (Gibco) according to the TissueLyser II (Qiagen) manufacturer's instructions. Cells debris was removed by centrifugation during 3 min at 8000 rpm in MiniSpin centrifuge (Eppendorf, Hamburg, Germany). Viral RNA was isolated from 100 µl of the obtained homogenates using a QIAamp Viral RNA Mini kit according to the manufacturer's instruction (Qiagen). cDNAs were synthesized using a Reverta-L kit (InterLabService, Moskow, Russian Federation) according to the manufacturer's protocol followed by RT-PCR using AmpliSens Influenza virus A/B-FL kit (InterLabService). For detection an infectious virus in organ's homogenates the $TCID_{50}$ assay was performed.
